# Judgment and Embodied Cognition of Lawyers. Moral Decision-Making and Interoceptive Physiology in the Legal Field

**DOI:** 10.3389/fpsyg.2022.853342

**Published:** 2022-03-24

**Authors:** Laura Angioletti, Federico Tormen, Michela Balconi

**Affiliations:** ^1^International Research Center for Cognitive Applied Neuroscience (IrcCAN), Università Cattolica del Sacro Cuore, Milan, Italy; ^2^Research Unit in Affective and Social Neuroscience, Department of Psychology, Università Cattolica del Sacro Cuore, Milan, Italy

**Keywords:** legal reasoning, moral decision-making, lawyers, ultimatum game, interoception

## Abstract

Past research showed that the ability to focus on one’s internal states (e.g., interoceptive ability) positively correlates with the self-regulation of behavior in situations that are accompanied by somatic and/or physiological changes, such as emotions, physical workload, and decision-making. The analysis of moral oriented decision-making can be the first step for better understanding the legal reasoning carried on by the main players in the field, as lawyers are. For this reason, this study investigated the influence of the decision context and interoceptive manipulation on the moral decision-making process in the legal field gathering the responses of two groups of lawyers. A total of 20 lawyers were randomly divided into an experimental group (EXP), which was explicitly required to focus the attention on its interoceptive correlates, and a control group (CON), which only received the general instruction to perform the task. Both groups underwent a modified version of the Ultimatum Game (UG), where are presented three different moral conditions (professional, company, and social) and three different offers (fair, unfair, and equal). Results highlighted a significant increase of Acceptance Rate (AR) in those offers that should be considered more equal than fair or unfair ones, associated with a general increase of Reaction Times (RTs) in the equal offers. Furthermore, the interoceptive manipulation oriented the Lawyers toward a more self-centered decision. This study shows how individual, situational, contextual, and interoceptive factors may influence the moral decision-making of lawyers. Future research in the so-called Neurolaw field is needed to replicate and expand current findings.

## Introduction

While moral choice behavior has received much attention in economics and psychology, it is rarely considered in the decision-making process applied to law. However, investigations into moral, regulatory, and decision-making judgments regarding persons involved in judicial proceedings are becoming more and more common in literature, while there are not many investigations regarding the main actors of justice and law, such as judges and lawyers (Goodenough and Zeki, 2006; Danziger et al., 2011; Tormen, 2020).

The analysis of the cognitive processes that lead to regulatory and legal reasoning by legal professionals has fundamental importance. And it is precisely the analysis of moral-oriented decision-making process that is the first step to better understand the legal reasoning carried out by the main players in the field, as lawyers are. Recently, a consensus view has emerged, which recognizes important roles for emotion and intuition, and which suggests that normative judgment is a distributed process in the brain (Goodenough and Prehn, 2004).

It was previously assumed that decision-making informed by the legal norm, as an expression of normative morality within a given culture, must necessarily be informed by cognitive processes strongly influenced by emotional components (Haidt, 2001). To better comprehend the moral decision-making process, the context of the Ultimatum Game (UG) permits us to investigate what is considered one of the pillars of human morality: fairness. Fairness is chiefly investigated in the context of the UG, an extensively studied game in psychology, neuroscience, philosophy, and behavioral economics (Nobandegani et al., 2020). The UG has a simple design: two players, the proposer and the responder, have to agree on how to split a sum of money. Proposer makes an offer. If the responder accepts, the deal goes ahead; if the responder rejects, neither player gets anything. In both cases, the game is over.

Current findings on embodied cognition support the view that the body and the mind are inextricably linked in the production of cognitions (Häfner, 2013). According to embodied cognition theories, higher cognitive processes entail reactivations of sensory-motor states that occur during the experience with the world (Pace-Schott et al., 2019). Similarly, emotional experience and cognitive functioning are strongly linked to the activation of interoceptive representations and meta-representations of body signals that promote interoceptive awareness (Herbert and Pollatos, 2012; Angioletti and Balconi, 2020; Balconi and Angioletti, 2022). Also, the neuroanatomic basis of interoception constitute the correlate of the body in the mind and the mechanisms that allow affective and cognitive activities to be embodied (Craig, 2002).

To investigate the deeper dynamics of moral decisions, this study highlights the value of interoception as a factor that influences the decision-making process also in the legal field. The literature suggests a relation between the rationality of decision making and the interoception construct (Sugawara et al., 2020), conceived as the perception of afferent information that arises from any point within the body, and which is transmitted to the brain (Craig, 2002). Specifically, individual differences in the accuracy of perceiving bodily interoceptive signals have been associated with affective and decision-making processing (Sugawara et al., 2020).

The relationship between decision-making processes and body correlates has been studied before. For instance, Somatic Marker framework of Damasio (1996) illustrated that increased skin conductance, reflecting sympathetic nervous activity (a main interoception pathway) preceded rational decision-making processes. Notably, participants with interoceptive dysfunction tend to select the disadvantageous option in a classical decision-making paradigm (Werner et al., 2009); in contrast, participants with increased interoceptive accuracy were likely to exhibit adaptive intuitive decision making (Dunn et al., 2010). Regarding moral decision-making in the UG, a previous study showed that experimental exposure to interoceptive signals influences participants’ behavior at the task. It was found that listening to one’s heart sound, compared to the other bodily sounds: (1) increased subjective feelings of unfairness, but not rejection behavior, in response to unfair offers and (2) increased the unfair offers while playing in the proposer role (Lenggenhager et al., 2013).

However, what is interesting to note is that Interoceptive Attentiveness (IA), i.e., attention focused on a particular interoceptive signal for a certain time interval (Schulz, 2016; Tsakiris and De Preester, 2018), is not a static dimension, but rather it can be manipulated and trained (Farb et al., 2013).

In the UG, usually, the rejection of asymmetric rewards is often seen as an important way for enforcing social norms and encouraging cooperative behavior (Fehr and Gächter, 2002). Past research showed that the ability to focus on ones’ internal states positively correlates with the self-regulation of behavior in situations that are accompanied by somatic and/or physiological changes, such as emotions, physical workload, and decision-making (Herbert et al., 2007; Werner et al., 2009; Dunn et al., 2010).

Another perspective demonstrated that interoception could render individuals more empathic toward others with greater emotional arousal and affect sharing (Grynberg and Pollatos, 2015). In this regard, a previous study demonstrated that specific categories of individuals, such as meditators, seems not to experience the acceptance of unfair offers as social norm violations, as suggested by their higher acceptance rates (ARs) for asymmetric offers at the UG, but more as an acceptance of the interoceptive qualities that accompany any reward (small or large) compared to nothing (Kirk et al., 2011). Indeed, in the study meditators were better able to maintain the focus on their internal bodily states and uncouple negative emotional responses while confronted with asymmetric offers, with a related activation of brain areas involved in attention to the present moment and interoception (Kirk et al., 2011).

Concerning the fairness of the moral decisions, previous findings suggest an increase in Reaction Times (RTs) for fair and unfair offers compared with equal ones in the company and prosocial conditions (Balconi and Fronda, 2020). This result was interpreted according to the social context and attributed to the indirect involvement of individuals’ interest in the company and prosocial conditions, for which equal offers (i.e., the offers in perfect balance, without concessions to the other) appear to be the most immediately acceptable options compared with fair and unfair ones, because they maintain an advantageous equilibrium, without gains or losses for anyone (Balconi and Fronda, 2020).

Given these premises, this exploratory study aims to explore if the modulation of the attention to internal states, namely IA, could influence the moral decision-making of lawyers at the UG. The experimental group of lawyers was explicitly required to focus the attention on their interoceptive correlates while performing the task, compared to the control group of lawyers, which were instructed to perform the task only.

The behavioral effects related to RTs and the total number of accepted responses found in the previous study of Balconi and Fronda (2020) are expected in the sample of lawyers involved in the present study. Specifically, higher RTs are expected for offers implying higher cognitive control and cognitive dissonance, and in conditions requiring the evaluation of self-interest. Moreover, higher acceptance of the fair altruistic offers (i.e., the offers where I give up something for the other) is expected for social and professional conditions, displaying empathic responses toward the others, compared to company condition, in which the self-interest dimension could emerge instead, as in the previous study (Balconi and Fronda, 2020). Finally, regarding the manipulation of IA, it is supposed that the experimental group of lawyers will display a “gain effect” by accepting more the rewarding offers, according to Kirk et al. (2011) evidence on meditators, compared to the controls. To the best of our knowledge, this is the first time IA manipulation has been applied to a specific sample of legal professionals, while performing a moral decision-making task.

## Materials and Methods

### Participants

Twenty lawyers with an age range between 25 and 54 years old took part in the present study. Participants were recruited on a voluntary basis; they were all physically health, Caucasian lawyers, mainly senior associates of a law firm. The following inclusion criteria were used for all participants: normal or corrected to normal visual acuity and absence of neurological or psychiatric pathologies. Exclusion criteria were age < 25 years old; less than 1 year of legal practice; the presence of neurocognitive deficits and a clinical history of neurological or psychiatric disorders. They were randomly assigned to experimental (EXP) and control (CON) group conditions; groups were matched for gender and age. All participants signed the informed consent and did not receive any compensation for their participation in the study. The research was conducted following the principles and guidelines of the Helsinki Declaration and was approved by the local ethics committee of the Department of Psychology of Catholic University of the Sacred Heart, Milan, Italy.

### Experimental IA Manipulation

Before the task, the CON group received the general instruction to perform the task, without the IA manipulation, while the EXP group was explicitly asked to concentrate on their interoceptive correlates, while observing the stimuli and received the following instruction: “*During this task, we ask you to focus your attention on your bodily sensations (such as on your breath). Try to observe how you feel and if there are any variations in your body as you perform the task*” (Balconi and Angioletti, 2021a,b).

### Moral Decision-Making Task

Participants were asked to perform a modified version of the task adopted by Balconi and Fronda (2019, 2020), implemented using the Qualtrics XM platform (Qualtrics LLC, Provo, UT, United States). The task, which consists of a modified version of the UG (Balconi and Fronda, 2019, 2020), proposed three different randomized moral conditions of choice (professional fit, company fit, and social fit) adapted to the legal context.

In particular, the task required two players: the proponent (different according to the context of choice) and the respondent (the individual who performed the task) to distribute a sum of money. The proposer decided how to distribute the sum of money, and the respondent could decide whether to accept or reject the proposed offer. If the respondent decided to refuse the offer, no player would take the money.

In the professional fit condition, the decision on how to divide the amount of money relates to one’s profession (e.g., lawyers were required to accept or reject the proposal of a colleague for a work done together). In the company fit condition, the decision on how to divide the sum of money concerns the effects on the business organization (e.g., lawyers were asked to accept or reject the proposal of the law firm for the realization of certain common benefits, such as the laundry service, in the Firm). In the social fit condition, the decision on how to divide the sum of money concerns the social context (e.g., lawyers were required to accept or reject the proposal of the law firm for making a financial contribution to a colleague’s relative with health problems).

For each condition (professional fit, company fit, and social fit), 10 coherent scenarios were presented. The different choice conditions were presented in three blocks of three randomized scenarios and each block lasted approximately 15 min. At the end of each scenario presentation, three different offers of distribution of money were proposed: equal (50% to the proposer and 50% to the responder), fair (60% to the proposer and 40% to the responder), and unfair (40% to the proposer and 60% to the responder).

Participants could accept or reject the proposed offer by clicking the “Accept” or “Reject” button on the survey. Specifically, the three offers (fair, unfair, and equal) were presented separately on the screen until the participant decided whether to accept or reject the offer proposed to record the response times. Moreover, participants were not given a defined time interval to decide whether to accept or reject the proposed offer.

**Figure 1:**
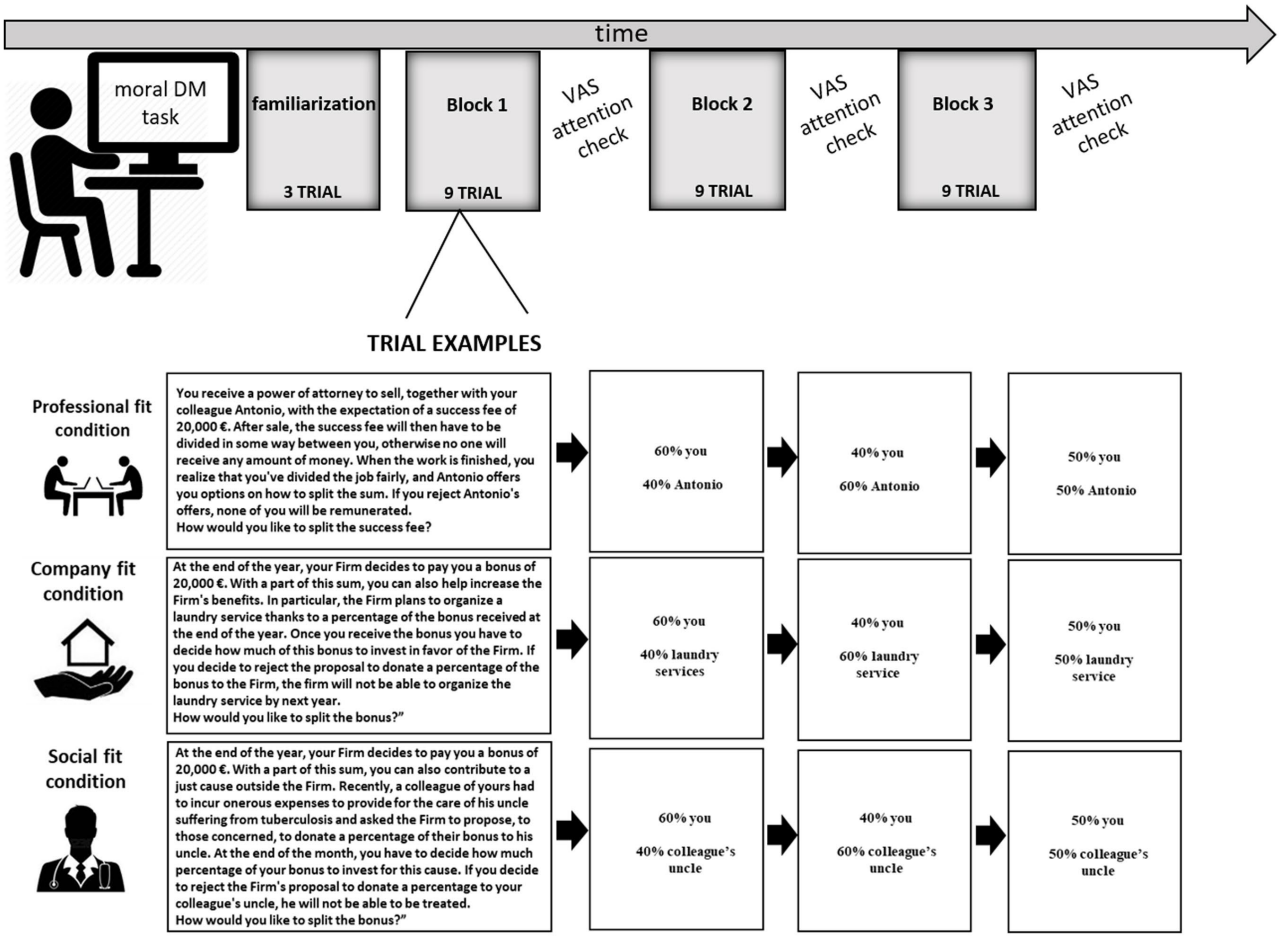
The research experimental procedure and sample trials.

After each block of three randomized scenarios, participants evaluated how much attention (i.e., their Attention Focus, AF) they paid to the situation, the self, or other on a Visual Analog Scale (VAS) ranging from 0 (no attention) to 10 (complete attention). This way, fluctuations of attention over the three blocks were assessed. For a graphical representation of the procedural steps and examples of scenarios, see Figure 1.

### Data Analysis

Visual Analog Scale scores, AR (total number of accepted proposed offers), and RTs for each condition related to participants’ choices were obtained. A first mixed repeated measure ANOVA with independent factors *Block* (3: First, Second, and Third) × *AF* (3: Situation, Self, and Other), and as between factor, the *Group* (2: EXP, CON) was applied to VAS scores. Then, two mixed repeated measures ANOVA with independent within factors *Condition* (3: Professional, Company, and Social) × *Offer Type* (3: Fair, Unfair, and Equal), and as between factor the *Group* (2: EXP, CON) were applied to behavioral measures, i.e., RTs and AR. For all the ANOVA tests, the degrees of freedom have been corrected using Greenhouse–Geisser epsilon where appropriate. The threshold for statistical significance was set to *α* = 0.05. Pairwise comparisons were applied to the data in case of significant effects. Simple effects for significant interactions were further checked *via* pairwise comparisons, and Bonferroni correction was used to reduce multiple comparisons’ potential biases. Furthermore, the normality of the data distribution was preliminarily assessed by checking kurtosis and asymmetry indices. The size of statistically significant effects has been estimated by computing partial eta squared (*η*^2^) indices.

## Results

### Visual Analog Scale Score

**Figure 2:**
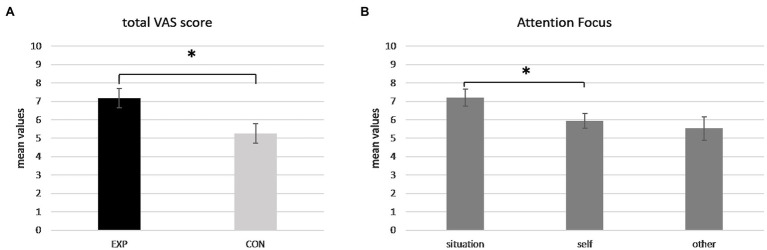
**(A,B)** Visual Analog Scale (VAS) score. Bar chart shows VAS scores in terms of **(A)** higher mean values of attention scores for the experimental compared to control group; **(B)** higher mean values of self-reported attention for the situation compared to the self. For all charts, bars represent ±1 SE; all asterisks mark statistically significant differences, with *p* ≤ 0.05.

ANOVA showed a significant main effect for *Group* [*F*(1,18) = 6,675, *p* = 0.019, *η*^2^ = 0.271]. Pairwise comparisons revealed higher mean scores for the EXP group compared to the CON group (*p* = 0.019; Figure 2A).

Secondly, a significant main effect for *AF* was detected [*F*(2,36) = 4,232, *p* = 0.041, *η*^2^ = 0.190]. In particular, pairwise comparisons showed significant higher mean scores for the situation compared to the self (*p* = 0.021; Figure 2B).

### Acceptance Rate

**Figure 3:**
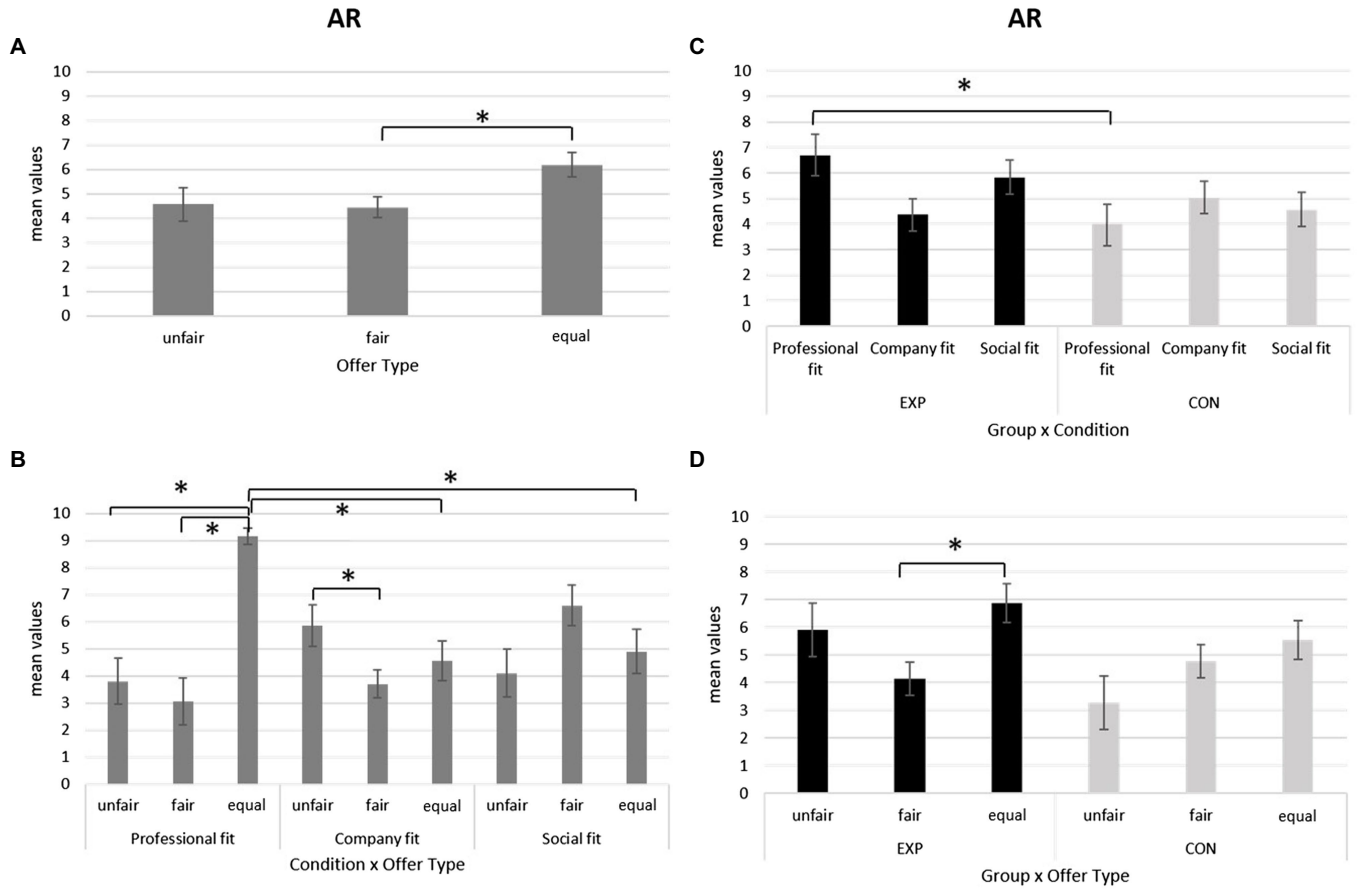
**(A–D)** Acceptance Rate (AR). **(A)** Bar chart shows higher Reaction Times (RTs) for equal compared to fair and unfair offers for the whole group of lawyers. **(B)** Bar graph displays higher AR for equal offers compared to fair offer for all participants. **(C)** Bar chart shows that in the professional fit condition there is an increase of accepted responses for the equal offers compared to unfair and to fair offers. Equal offers were more accepted in the professional fit condition compared to the company and social fit condition. In the company fit condition, higher values of accepted response were found for the unfair compared to the fair offers. Fair offer type was more accepted in the social fit condition than in the professional fit and in the company fit condition. **(D)** Bar graph displays higher accepted responses in the professional fit condition for the experimental group (EXP) compared to the control (CON) group. For all charts, bars represent ±1 SE; all asterisks mark statistically significant differences, with *p* ≤ 0.05.

For individuals’ options of response, ANOVA revealed a significant main effect for Offer Type [*F*(2,36) = 4.977, *p* = 0.014, *η*^2^ = 0.217]. Pairwise comparisons showed a significant increase of accepted responses for equal offers compared to fair offers (*p* = 0.037; Figure 3A).

Secondly, ANOVA showed a significant interaction effect *Condition* × *Offer Type* [*F*(4,72) = 13.809, *p* < 0.001, *η*^2^ = 0.434]. Specifically, simple effect analysis revealed that in the professional fit condition there is an increase of accepted responses for the equal offers compared to unfair ones (*p* < 0.001) and, also, compared to fair offers (*p* < 0.001). Instead, in the company fit condition, higher values of accepted response were found for the unfair offers compared to the fair offers (*p* = 0.029).

In addition, simple effect analysis revealed that fair offer type is more accepted in the social fit condition than in the professional fit (*p* = 0.045) and in the company fit condition (*p* = 0.003). Moreover, equal offers were more accepted in the professional fit condition compared to the company fit condition (*p* < 0.001) and social fit condition (*p* < 0.001; Figure 3B).

Thirdly, a significant interaction effect *Condition* × *Group* was detected [*F*(4,72) = 6.342, *p* = 0.013, *η*^2^ = 0.261]. As revealed by pairwise comparisons, an increase of accepted responses in the professional fit condition was found for the EXP group compared to the CON group (*p* = 0.030; Figure 3C).

Lastly, a significant interaction effect *Offer Type* × *Group* was found [*F*(4,72) = 3.546, *p* = 0.043, *η*^2^ = 0.165]. In particular, the pairwise comparison showed that for the EXP group significantly higher accepted responses were obtained for equal compared to fair offers (*p* = 0.020; Figure 3D).

### Reaction Times

**Figure 4:**
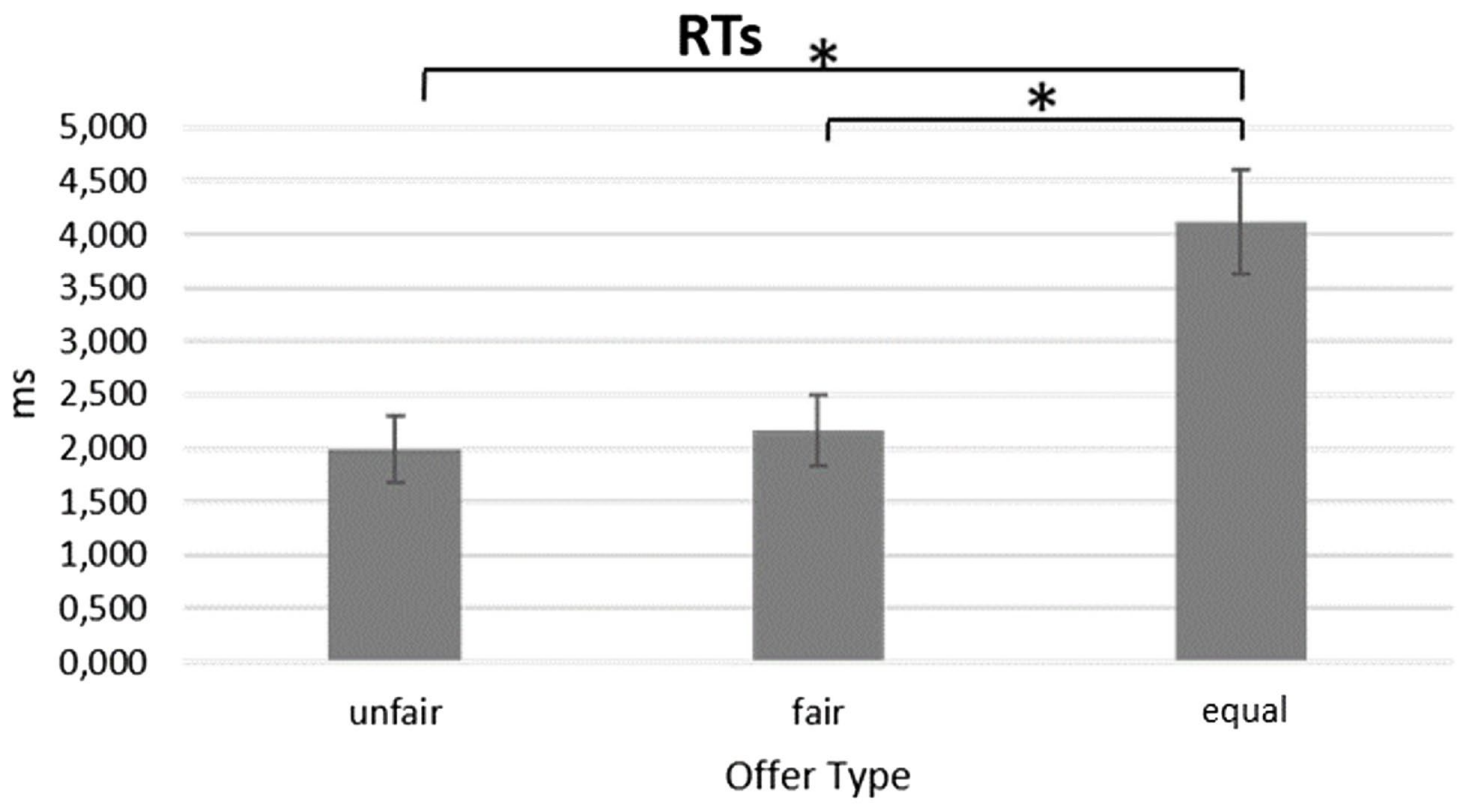
Reaction Times. Bar chart shows higher AR of equal compared to fair offers in the EXP group. Bars represent ±1 SE; all asterisks mark statistically significant differences, with *p* ≤ 0.05.

For the RTs, a significant main effect for *Offer Type* was found [*F*(2,36) = 17.221, *p* < 0.001, *η*^2^ = 0.489]. The pairwise comparison revealed significantly lower RTs for unfair offers compared to equal offers (*p* < 0.001). Also, the pairwise comparison showed significantly lower RTs for fair offers compared to equal offers (*p* = 0.002; Figure 4).

## Discussion

This exploratory study investigated lawyers’ moral choices concerning different decision-making conditions and offers within a law firm context. The manipulation of the IA during the presentation of different moral scenarios and offers made it possible to investigate behavioral responses in relation to three moral decision-making conditions (professional fit, company fit, and social fit) and offers (fair, unfair, and equal).

Firstly, we have observed a general increase of AR for equal offers than fair or unfair ones. This general evidence shows how lawyers are more willing to offer benefits equally, despite this choice having a noticeable cost in terms of RTs. As highlighted in the results, it seems that being quicker in selecting both fair and unfair options compared to equal options, independently from the condition, constitutes easier choices for lawyers. Moreover, a general increase of RTs in the equal decisions was observed: all the lawyers spent more time for accepting an equal offer compared to the other offers proposed.

Previous literature showed that cognitively complex processes and conflicts restrain economically self-interested responses (Halali et al., 2011) require higher cognitive cost and resources which are associated with higher RTs; on the contrary, cognitively fewer complex processes are associated with faster RTs, as they require less information processing (Klapp, 2010).

Therefore, in the present study, regardless of the condition, a possible interpretation could be that lawyers were more likely to make immediate fair and unfair choices (significant reduction of RTs for both fair and unfair offers), perhaps due to less complex cognitive processes and because of the higher direct engagement, which supports a more immediate ability to produce the moral decision. On the other hand, it may be possible that equal choices required a greater cognitive decision-making effort for lawyers, perhaps because of the greater degree of uncertainty in the choice, due to the assessment that does not directly concern one’s interests.

This result is partially in contrast with a previous study showing an increase of RTs for fair and unfair offers compared with equal ones, specifically in the company and social fit conditions (Balconi and Fronda, 2020). However, two main aspects distinguish our result on RTs from the evidence of this previous research and are that (i) the variation in RTs was interpreted in relation to the condition in which the offers were significantly accepted (company and social fit condition), and (ii) the study referred to a sample of managers, not including lawyers. The lawyers’ category may differ, in terms of moral decision-making behavior, from other professional categories, and therefore it would be interesting for future studies to test the differences between different professional groups, even for clarifying the present results.

Secondly, the AR effect for equal offers was mainly found in the professional condition, in which the whole sample of lawyers tend to accept equal than fair and unfair offers more frequently. While in the company fit condition, lawyers accept more unfair offers compared to fair offers. Specifically, it is plausible that a rational responder motivated purely by self-interest prefers to accept the equal amount of money offered by the proposer (i.e., the colleague, in the professional condition), as this offer will represent a fair gain for a work task equally done together. Instead, lawyers were more willing to accept unfair offers for deriving personal benefits when faced with splitting the sum of money with the law firm, in the company condition. In line with the previous study (Balconi and Fronda, 2020), in which the abovementioned sample of managers tend to accept more personally advantageous offers when in the company condition, a possible explanation could be that, even for the present group of lawyers, the company condition has been conceived as a distant or “external” situation, not personified, and therefore less relevant on a personal level (where individuals can gain some advantages without excessive “moral costs”). As previously suggested by Balconi and Fronda (2020), when moral decision-making dynamics are framed by the company context, the decision process seems to be strongly influenced by the subjective understanding of the inherent benefits to themselves, more than by the disadvantages for the others, intended as the company or, in this case, as the law firm.

Thirdly, the evaluation linked to the benefits and advantages of others, also compared to one’s advantages, varies depending on the decision context (professional, company, or social fit conditions). As highlighted above, in the company condition lawyers preferred the unfair compared to fair offers, whereas in the social condition fair offers are more accepted compared to the professional and company condition. Furthermore, in the professional condition, the equal offer is significantly more accepted than in the company and social condition. Therefore, the condition in which offers are proposed may modulate the lawyers’ moral decision-making process, since they seem to be more unfair in a company condition, fairer in a social condition, and, most of all, equal in a condition of professional engagement with a colleague.

A possible explanation for these outcomes could be that lawyers could prefer to accept offers that are inherently more advantageous and promote their self-interest in decisions involving the law firm, perhaps displaying less empathic and altruistic behavior. In contrast, lawyers showed more empathic behavior in the conditions, where other individuals such as the relative of a colleague (the social condition) are involved in the attribution of the sum of money. Indeed, as shown by some previous research, empathic behavior can facilitate a greater understanding of the potential effects, consequences, and obligations of actions about the well-being of others and allows to better evaluate the cost of personal choices and the extent of social benefits (Mencl and May, 2009; Dietz and Kleinlogel, 2014). As supposed in our hypotheses, it is possible that in the social fit condition, lawyers experienced the highest levels of empathy with respect to the company and professional fit condition and they tend to overshadow their self-interest for pursuing a more important social cause.

Fourthly, as highlighted in the results of VAS scores, the manipulation of the attention to the internal state induced in the lawyers increased subjective attention to the situation concerning the self or other conditions. Moreover, the EXP group, which was explicitly required to focus the attention on their interoceptive correlates while performing the task, significantly expressed higher general levels of attention during the task compared to the CON group. The present finding could be considered the first evidence of the successful manipulation of the interoception in the EXP group of lawyers. Despite this interesting preliminary result and the use of VAS in previous similar studies (Lenggenhager et al., 2013), we are aware it consists of a subjective self-report measure of attention and further studies would benefit from the integration of objective measures, applied both before (e.g., heartbeat detection task, to control individual differences in interoceptive ability) and during the task (e.g., eye-tracking technique, as an indirect measure of the attentional focus; cortical frequency bands recording through electroencephalogram).

Regarding the different moral conditions in the task, the EXP group showed higher AR for offers displayed in the professional fit situation than the CON group. This result could be related to the fact that the interoceptive manipulation oriented the lawyers’ focus toward a more self-centered decision process, which aims to obtain a direct greater profit for themselves. It is worth noting the professional condition is the unique condition in which participants were explicitly involved in the first person compared to the other conditions (since they were required to split a sum of money about work they conducted together with a colleague). In line with this, previous research found that receiving interoceptive feedback might enhance self-centered perspective taking and “first-person perspective” (Kirk et al., 2011; Lenggenhager et al., 2013).

Regarding the type of offer, it seems that regardless of the condition, the EXP group preferred more equal offers than fair and unfair ones, at the expense of personal and others’ advantage. Perhaps, a possible explanation for this second effect is that IA in this group might enhance a more equal attitude, with a preference for equal and more balanced choices. This result is partially in contrast with study of Kirk et al. (2011), for which specific groups that are trained to modulate their IA (i.e., meditators) tend to show higher acceptance rates for asymmetric offers at the UG, but also with Lenggenhager et al. (2013) and with Piech et al. (2017) research for which only interoceptive sensitivity, conceived as a trait component, predict altruistic behavior in the dictator game. In the literature, the ability to accurately detect one’s internal body signals has been also associated with cognitive and emotional components of empathy, in terms of greater emotional arousal and affect sharing toward others (Grynberg and Pollatos, 2015). Therefore, future studies are needed to better deepen this “equity effect” in lawyers and the basic relation between IA and moral decision-making in the UG.

To the best of our knowledge, there is still no evidence in the literature about this specific phenomenon, and no previous data or analysis regarding the lawyers were reported in similar studies. Indeed, despite the innovativeness of the paradigm, the present exploratory study has some limitations: the sample size should be augmented and a multimethodological approach should be adopted to collect also the neural and psychophysiological correlates underlying the cognitive and emotional moral decision-making processes (Balconi and Molteni, 2016). Therefore, future research is needed to replicate and expand current findings.

## Data Availability Statement

The datasets used and/or analyzed during the current study are available from the corresponding author on reasonable request.

## Ethics Statement

The studies involving human participants were reviewed and approved by Department of Psychology, Catholic University of the Sacred Heart, Milan, Italy. The patients/participants provided their written informed consent to participate in this study.

## Author Contributions

LA and FT wrote the first draft and each section of the manuscript. LA, FT, and MB contributed to the manuscript final writing and revision, read, and approved the submitted version.

## Conflict of Interest

The authors declare that the research was conducted in the absence of any commercial or financial relationships that could be construed as a potential conflict of interest.

## Publisher’s Note

All claims expressed in this article are solely those of the authors and do not necessarily represent those of their affiliated organizations, or those of the publisher, the editors and the reviewers. Any product that may be evaluated in this article, or claim that may be made by its manufacturer, is not guaranteed or endorsed by the publisher.
